# Semicircular Canals Input Can Modify the Fast-Phase Nystagmus in Off-Vertical Axis Rotation of Mice

**DOI:** 10.1523/ENEURO.0461-24.2025

**Published:** 2025-03-04

**Authors:** Shijie Xiao, Tong Zhao, Wenda Liu, Zihao Peng, Fangyi Chen

**Affiliations:** ^1^Department of Biomedical Engineering, Southern University of Science and Technology, Shenzhen 518055, China; ^2^Giant Technologies Co., Ltd, Shenzhen 518055, China

**Keywords:** fast-phase nystagmus, off-vertical axis rotation, slow-phase nystagmus, unilateral vestibular lesion, vestibular ocular reflex, vestibular organ

## Abstract

Vestibular research is essential for understanding and treating disorders such as vertigo and Meniere's disease. The vestibulo-ocular reflex (VOR) is a key method for assessing vestibular function and an essential tool for diagnosing vertigo. Traditionally, the VOR comprises angular VOR (aVOR) and translational VOR (tVOR), which originate from the vestibular semicircular canals (SCCs) and otolith organs, respectively. VOR consists of both fast-phase and slow-phase eye movements, which functionally interact to contribute to gaze control. However, to calculate the gain and phase parameters of the VOR, it is common practice to exclude fast-phase information superimposed on slow-phase eye movements. As a result, the information contained in the fast phase has not been fully utilized. OVAR is primarily used to evaluate otolith function, as there is no SCC input during its steady state. It is widely accepted that fast-phase nystagmus (FPN) during OVAR is generated by periodic otolith inputs via the central vestibular velocity storage mechanism. Surprisingly, we discovered in this study that SCC input can modify the generation of FPN in mouse OVAR test, as demonstrated by testing *Zpld1* (Zona pellucida-like domain containing 1 protein) mutant mice with SCC deficits. This finding was further confirmed using both unilateral and bilateral semicircular canals dehiscence surgical models. In addition to revealing the dependence of FPN on SCC input, we demonstrated that FPN can be used to evaluate vestibular function, particularly in conditions that are difficult to assess using slow-phase eye movements, such as unilateral vestibular lesions and central modulation via baclofen treatment.

## Significance Statement

Although the vestibular semicircular canal (SCC) input is absent during the steady state of OVAR test, we discovered that SCC input can modify the generation of fast-phase nystagmus (FPN) in mice. This was demonstrated using *Zpld1* mutant mice with SCC deficits and further confirmed through semicircular canal dehiscence models. Additionally, we found that FPN is valuable for assessing vestibular function in conditions such as unilateral vestibular lesions and in cases of central modulation by baclofen, making it a promising diagnostic tool for vestibular clinics.

## Introduction

Vestibular research is essential for understanding and treating disorders such as vertigo, Meniere's disease, and vestibular migraines, which are prevalent in aging populations and increase the risk of falls (Osoba et al., 2019). It also plays a key role in space exploration and addresses vertigo and vomiting induced by virtual reality (Ng et al., 2020; Hupfeld et al., 2022). In recent years, mice have become the predominant model in inner ear research and are widely used in studies on vestibular toxicity (Zeng et al., 2020; Gao et al., 2022), development (Burns and Stone, 2017), genetics (Armstrong et al., 2015; Vijayakumar et al., 2019; Simon et al., 2021), and function. Vestibular function is a crucial indicator for understanding and monitoring the physiological state of the vestibular system, highlighting the urgent need for effective vestibular function assessments in these research areas. The vestibulo-ocular reflex (VOR) originates from the peripheral vestibular end organs including vestibular semicircular canals (SCCs) and otolith organs and transmits signals to the extraocular muscles through a direct or relatively direct pathway in the brainstem (Katsarkas et al., 1991), where head velocity information is converted into appropriate oculomotor signals (Katsarkas et al., 1991). Furthermore, the VOR is also modulated by the central nervous system to facilitate vestibular compensation or adaptation. Recently, with the development of both customized (Nist-Lund et al., 2019; Ono et al., 2020) and commercially available VOR test systems (Yang et al., 2020), mouse VOR tests have gradually surpassed the previously prevalent method of vestibular sensory evoked potentials (VsEP) and have become more popular in various laboratories.

It is important to note that, VOR contains both fast-phase and slow-phase eye movements (Furman, 2010), which interact to contribute to gaze control. However, to calculate the gain and phase parameters of the VOR, it is common practice to exclude fast-phase information superimposed on the slow-phase eye movements (Ebisawa et al., 1988; Ranjbaran et al., 2016; Kim et al., 2021), leaving the information in fast-phase nystagmus (FPN) underutilized (Katsarkas et al., 1991; Furman, 2010). Slow-phase eye movement is determined by the direct pathway of the angular VOR (aVOR) or the relatively direct pathway of the translational VOR (tVOR; Shimizu et al., 2016), and thus its features (gain and phase) are insufficient to reflect many subtle but important vestibular deficits involving the central pathway. In contrast, the generation of FPN depends on both direct and central pathways. Experiments have demonstrated that FPN is related to central VSM (Laurens and Angelaki, 2011; Shimizu et al., 2016), a regulatory mechanism involving the vestibular cerebellum, which is primarily responsible for establishing multisensory rotation signals (Laurens and Angelaki, 2011). Since both peripheral and central systems are involved in generating FPN, abnormal sensory signal processing can arise from either central or peripheral vestibular inputs (Choi et al., 2007). Therefore, efficiently utilizing FPN may be beneficial for detecting vestibular deficits, especially those that are undetectable by slow-phase eye movements. Furthermore, FPN in vision-induced eye movement has been used to study the central nervous system disorders (Kanayama et al., 1987) and the effect of psychiatric drugs (Wang et al., 2005). FPN in aVOR has been used to determine the lesion side in unilateral vestibular lesion (UVL; Curthoys, 2002). Thus, studying FPN not only helps in understanding the central integration of peripheral inputs but also in discovering new clinical applications.

The aVOR and OVAR are commonly used in clinical and animal experiments to assess the functionality of the vestibular peripheral SCCs (Beraneck and Cullen, 2007; Fetter, 2007) and otolith organs (Brettler et al., 2000; Maruta et al., 2001), respectively. In aVOR mode, sinusoidal yaw oscillation primarily stimulates the horizontal SCCs (H-SCCs). In contrast, during OVAR mode, constant speed rotation along a tilted axis results in continuous changes in head orientation relative to gravity. Eye movements during OVAR are complex, involving horizontal, vertical, and torsional components. During the steady state of OVAR, the input from SCCs ceases, and eye movements are primarily driven by otolith signals. Vertical and torsional sinusoidal modulation components (SMC) are mainly utilized for evaluating changes in vestibular function due to otolith stimulation along the interaural and naso-occipital axes (Angelaki and Hess, 1996). The horizontal SMC (H-SMC) is small and can be isolated by eliminating the strong FPN.

In this study, we investigated the horizontal FPN (H-FPN) in OVAR. Several reasons guided our focus on H-FPN in OVAR: (1) it reflects both central and peripheral conditions of the vestibular system; (2) H-FPN was prominent in horizontal eye movement in mouse OVAR test, an aspect that is often underutilized in the analysis of results. During our investigation of this overlooked feature, we found that the input from SCCs can modify the generation of H-FPN in mouse OVAR test, consistent with findings from primate studies. Moreover, H-FPN is sensitive to various vestibular deficits, highlighting its potential as a diagnostic tool in vestibular clinics.

We developed a four-channel mouse pseudo-OVAR (pOVAR) testing system based on the principle of eccentric counter-rotation (CR; Kaufman, 2002; Shimizu et al., 2015). This dual-axis setup generates separate or combined stimuli to the SCCs and otolith organs. Eccentric axis movement provides angular acceleration stimulation, while the main axis rotation applies linear force stimulation. When both axes rotate at a constant velocity, the system simulates the tilted rotation modes of OVAR. The FPN in the horizontal eye movements during this pOVAR stimulation was extracted to assess the vestibular dysfunction.

The system was validated using three vestibular deficit mouse models: (1) IDPN, a commonly used vestibular toxicant (Khan et al., 2009), which causes loss of inner ear hair cells in rodents, leading to irreversible damage to the peripheral vestibular system (Wilkerson et al., 2018; Zeng et al., 2020). IDPN is frequently employed in studies of inner ear hair cell regeneration. (2) Baclofen, a vertigo-control drug, acts as an agonist of gamma-aminobutyric acid type B (GABA_B_) receptors (Dai et al., 2006; Ebenezer and Prabhaker, 2007; Shimizu et al., 2016). GABA is a major neurotransmitter involved in the VSM, and baclofen can inhibit neuronal activity in this network, affecting vestibular function. Both drugs were administered intraperitoneally at various concentrations. The results demonstrated a strong correlation between FPN frequency and drug concentration. (3) UVL, a common model in vestibular research, simulates clinical conditions such as vestibular neuritis (Fetter, 2016) to study compensation and adaptation in the vestibular system (Sadeghi et al., 2011; Hubner et al., 2017; Khan et al., 2022). A critical challenge in UVL models is identifying the side of the lesion. During the early stages, the aVOR slow-phase eye movements are asymmetrical to the lesion side but gradually becomes symmetrical due to the central compensation of asymmetrical inhibition after ∼1–2 weeks. Although it is claimed that asymmetrical slow-phase aVOR can be detected (Beraneck et al., 2008; Hubner et al., 2017), results are often inconclusive, and it is generally accepted that the lesion side cannot be identified from slow-phase eye movements due to the bilateral vestibular “push–pull” mechanism and the strong compensatory ability of the vestibular system (Fetter, 2016). However, FPN, which involves the central pathways, can reflect asymmetrical inhibition by the central vestibular system. We demonstrated asymmetrical FPN frequency toward and away from the lesion side in UVL models.

The most intriguing finding was that mice with bilateral horizontal semicircular canal damage failed to produce FPN in the OVAR test. The *Zpld1^−/−^* mouse, which has impaired canal function but normal otolith function, exhibited a normal vertical SMC (V-SMC) during OVAR. However, the horizontal H-FPN was nearly absent, consistent with the OVAR results in monkeys that underwent bilateral H-SCC neurectomy. This finding was confirmed in both unilateral and bilateral semicircular canals dehiscence (SCD) models. The V-SMC was normal in unilateral SCD mice and mildly impaired in bilateral SCD mice, but H-FPN was significantly reduced in both cases. These results indicate that SCC inputs may modify the generation of H-FPN in the mouse OVAR test.

In addition to this discovery, with this system, experimental results demonstrated the advantage of utilizing FPN in effectively identifying various subtle vestibular deficits, especially those undetectable with traditional V-SMC in OVAR. Moreover, the structure can accommodate multiple eccentric rotation platforms within the same main rotation circle, thus enhancing spatial efficiency for constructing a high-throughput system.

## Materials and Methods

### The four-channel dual-axis mouse VOR test system

**Figure 1.**
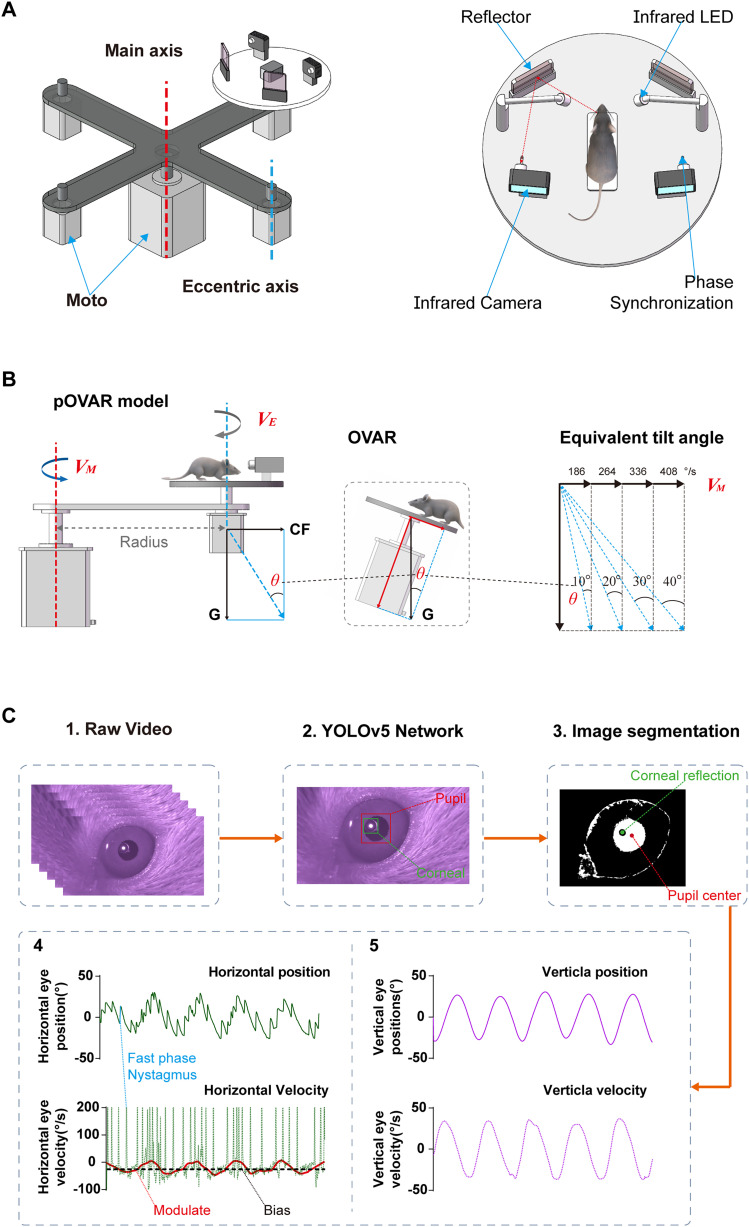
Four-channel mouse VOR test system. ***A***, Left, The schematic of four-channel system with one main axis (dashed red line) and four eccentric axes (dashed blue line). Right, Layout diagram of the turntable at the eccentric axis. Two infrared motion cameras are mounted on micromanoeuver platforms and record mouse eye movements via mirrors (dashed red line). The LED in front of the camera emits light for 10 ms when the turntable starts, which is used to calculate the VOR phase. ***B***, The pOVAR test mode. ***C***, Mouse eye movement video processing flowchart. CW, clockwise; CCW, counter clockwise; *V_M_*, main axis velocity; *V_E_*, eccentric axis velocity; G, gravity; CF, centrifugal force.

The diagram of the four-channel mouse VOR test system is shown in Figure 1*A*. Testing parameters (main and eccentric axis rotation velocity, direction, duration, etc.) can be programmed into a motor controller to manipulate the main and eccentric axis motor movement, thereby performing corresponding vestibular stimulation. The centers of the mouse binaural coincide with the center of rotation of the eccentric axis, ensuring alignment between the vestibular system and the rotation axis. Simultaneously, two infrared motion cameras (EZVIZ S6, China) record the eye movement trajectories of mice, with the videos transmitted to a computer via Wi-Fi for subsequent analysis.

The pOVAR modes were used in this study. In the conventional OVAR mode, the animal is tilted relative to the horizontal plane, generating roll and pitch of the head while rotating the platform, as shown in Figure 1*B*. In pOVAR mode, this tilt is simulated by the centrifugal force relative to the Earth's vertical axis, generated by the main axis rotation. The angle *θ* represents the angle of resultant gravity-centrifugal force relative to the Earth's vertical axis, equivalent to the tilt in the classic OVAR test. The magnitude of the simulated tilt angle *θ* is determined by the main axis rotation velocity and radius, considering a constant gravity of 1 g. For a fixed radius of 17 cm in our setup, the main axis velocities *V_M_* = 186, 264, 336, 408°/s simulates tilt angle *θ* = 10°, 20°, 30°, 40° in a conventional OVAR test. Meanwhile, the eccentric axis rotation velocity determines the modulation frequency of the linear acceleration (*V_E_* = 36, 72, 108, 144°/s corresponds to frequency *f* = 0.1, 0.2 0.3, 0.4 Hz). During steady-state operation of the system, the direction of the mouse VOR is related only to the direction of the eccentric axis rotation and is independent of the direction main axis movement.

### Stimulus protocols

The experiments were conducted in complete darkness to avoid visual interference. Mice were treated with 2% pilocarpine 10 min before the experiment to prevent pupil dilation in darkness. To thoroughly investigate the characteristics of the mouse vestibular system, two different rotation paradigms were employed.

#### pOVAR mode for testing otolith organs

Both the main and eccentric axis rotate simultaneously at their own constant velocity and four mice can be tested simultaneously (Fig. 1*C*). Two different rotation paradigms were performed during pOVAR: (1) at fixed main axis velocity (336°/s = 30°) with different rotational velocity of eccentric axis (72, 108, 144°/s = 0.2, 0.3, 0.4 Hz) and (2) at fixed eccentric axis velocity (72°/s = 0.2 Hz) with different main axis velocity (186, 264, 336, 408°/s = 10°, 20°, 30°, 40°). The rotational direction (CW or CCW) of the two axis was set according to the experimental needs (Fig. 3). To obtain sufficient eye movement information, a 3 min video was recorded under each parameter condition, with at least a 1 min interval between different parameter experiments. The evoked V-SMC was used to evaluate the otolith dysfunction. More interestingly, the FPN was used to show the contribution of the SCCs to this conventional otolith measurement method.

### Animals

All animal care procedures and experimental protocols described in this paper were approved by the Animal Care and Ethics Committee of the Southern University of Science and Technology. Mice were housed under 12 h light/dark cycling conditions with *ad libitum* access to water and food. The experiments involved both genders at 6–8 weeks of age, each weighing ∼20 g. Three mouse models, IDPN, baclofen, and UVL, were used to validate the system and demonstrate the potential of FPN in evaluating subtle vestibular deficits. Two genetic models, *zpld1* and *otop1* (Otopetrin1) mice, along with unilateral and bilateral SCD mice, were used to investigate the impact of the semicircular canal (SCC) on the generation of H-FPN during pOVAR.

#### Vestibulo-toxicity—IDPN administration

**Figure 2.**
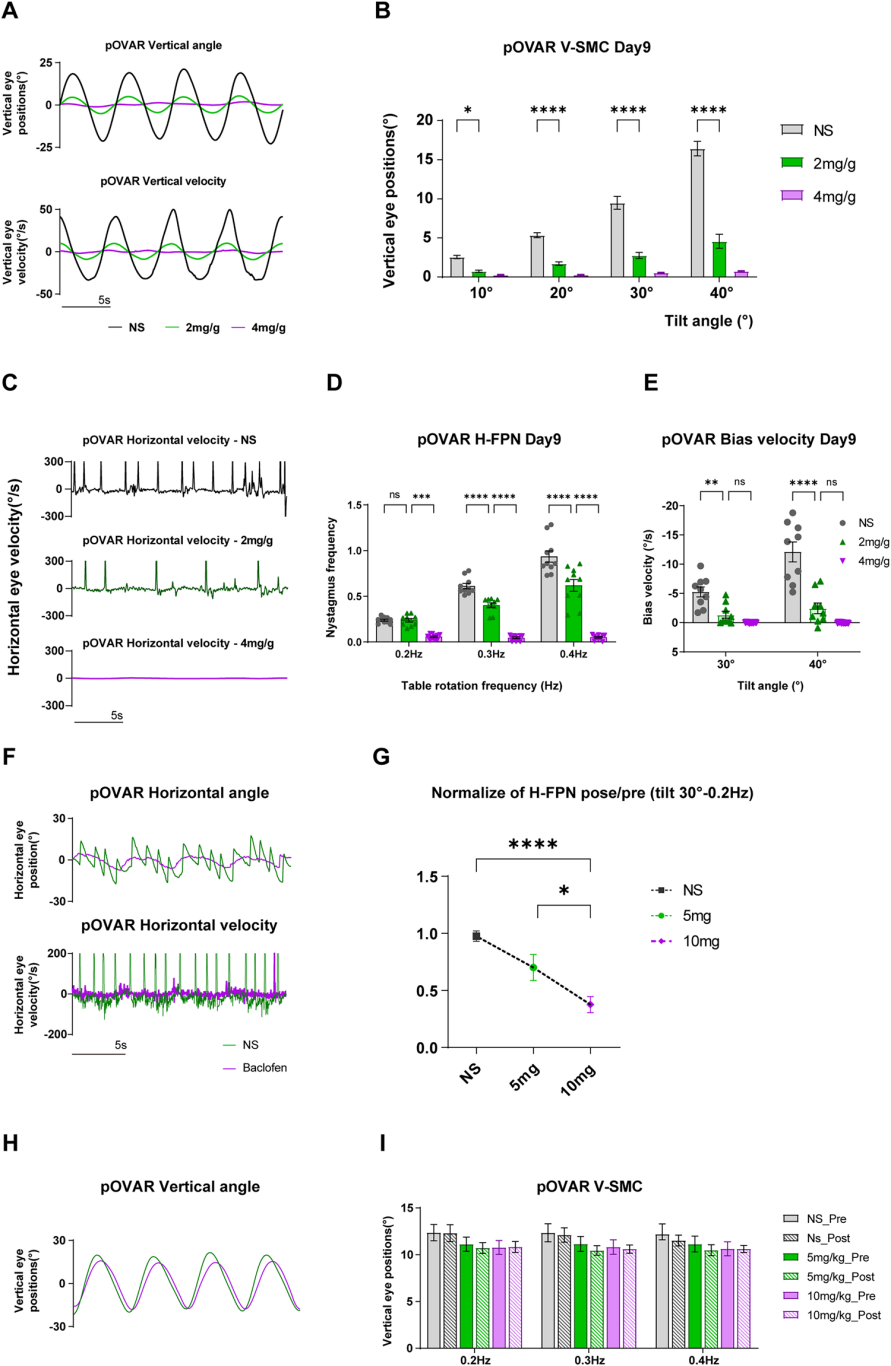
Effect of IDPN and baclofen on mouse vestibular function. Means ± standard error of mean (SEM) is shown in the Results. ***A***, Traces display vertical eye displacement and velocity in mice. ***B***, V-SMC results for the three groups on the Day 9 in the pOVAR test (tilt angle: 10°, 20°, 30°, 40° at 0.2 Hz). ***C***, Traces show horizontal eye velocity (tilt angle: 30°, 0.2 Hz) for control, mid-dose (2 mg/g), and high-dose (4 mg/g) groups. ***D***, The H-FPN frequency for three groups on the Day 9 of the pOVAR test (tilt angle: 30° at 0.2, 0.3, 0.4 Hz). ***E***, The horizontal mean slow-phase velocity for three groups on the Day 9 of the pOVAR test (tilt angle: 30°, 40° at 0.2 Hz in CW direction). CW is a positive direction, so the bias component is negative. ***F***, Traces display horizontal eye displacement and horizontal eye velocity in control and baclofen-treated groups. ***G***, The ratio of H-FPN frequency post-baclofen injection compared with pre-injection (tilt angle: 30° at 0.2 Hz). After administration, the H-FPN frequency significantly decreased in the high-dose group (10 mg/kg), while no significant change was observed in the control group. ***H***, Traces display vertical eye displacement in control and baclofen-treated groups. ***I***, pOVAR V-SMC results in the three groups (tilt angle: 30° at 0.2, 0.3, 0.4 Hz). Baclofen injection had little impact on SMC during pOVAR test (*p < 0.05, **p < 0.01, ***p < 0.001, ****p < 0.0001).

3,3′-Iminodipropionitrile (IDPN, TCI, I0010) was dissolved in physiological saline solution. Eighteen C57BL/6J wild-type mice were used for the vestibulotoxic drug study and divided into two experimental groups and one control group. Mice in the two experimental groups were injected intraperitoneally with IDPN at doses of 2 and 4 mg/g (≈4 µl/g), respectively. Mice in the control group received the same volume of saline solution (4 µl/g). The pOVAR test was conducted before and after IDPN injection (Fig. 2*A–D*).

#### Central vestibular manipulation—baclofen administration

Systemic administration of baclofen specifically inhibits VSM activity and decreases susceptibility to motion sickness (Dai et al., 2006; Cohen et al., 2008). Baclofen was purchased from MedChemExpress (MCE, HY-B0007) and dissolved in physiological saline solution. Fifteen C57BL/6J wild-type mice were divided into three groups: two experimental and one control. The experimental group mice received intraperitoneal injections of baclofen at a dosage of 5 and 10 mg/kg. Control group mice were injected with saline. The pOVAR test was conducted before and 90 min after drug administration. The dosage and administration route were based on previous studies (Ebenezer and Prabhaker, 2007; Shimizu et al., 2016).

#### Unilateral vestibular lesion

Surgical procedures for unilateral creating vestibular peripheral lesions and postoperative care are based on previously published methods (Guo et al., 2017, 2018). First, an adult mouse was anesthetized via intraperitoneal injection of dexmedetomidine hydrochloride injection (Dexdomitor, 0.08 mg/kg) and Zoletil 50 (32 mg/kg), while meloxicam (1 mg/kg) was administrated subcutaneously for analgesia. Next, the skin of mouse's head was disinfected with 75% ethanol, and an incision was made along the retroauricular groove to expose the lateral semicircular canal (LSC) and the posterior semicircular canal (PSC). A small hole was drilled in the middle of the LSC using a 26 G needle, allowing endolymph to leak. The hole was then enlarged slightly beyond the diameter of the injection cannula. The cannula was inserted ∼2 mm into the LSC toward the ampulla, and 15 µl of gentamycin (MACKLIN, G810322, 8 mg/ml) was injected using a microsyringe pump (TJ-2A). After the injection, the LSC hole was sealed with a small piece of muscle.

#### Unilateral and bilateral semicircular canals dehiscence

Injection of gentamicin through the semicircular canal can lead to damage to both the unilateral semicircular canal and the otolith organs. In contrast, the SCD technique selectively damages the semicircular canal while preserving the function of the otolith organs. Following the procedure for UVL, the SCD involves drilling a hole in the middle of the LSC, absorbing the leaking endolymphatic fluid with sterile cotton, and leaving the LSC hole unsealed. Performing unilateral or bilateral SCD surgery allows selective damage to the respective semicircular canals while preserving otolith function.

To account for surgical variability and individual differences in vestibular system performance, we increased the number of experimental mice to reduce error. Twenty-eight C57BL/6J wild-type mice were divided into three groups: 8 served as controls, 10 underwent left-UVL, and 10 underwent right-UVL. Mice were allowed 10 d to recover postoperatively, and the pOVAR test was conducted before and after surgery.

### Mice with genetic deficits in SCC and otolith organ

*Otop1* and *Zpld1* mutant mice were used for validating the system performance in localizing vestibular organ dysfunction. The *Zpld1* mouse was also used to investigate the necessity of SCC input for the H-FPN in the pOVAR test mode. The spontaneous recessive mutation in the Otop1 gene, located on chromosome 5, alters the pH of the inner ear, preventing the development of otolith particles composed of calcium carbonate crystals (Hurle et al., 2003). Consequently, *Otop1^tlt/tlt^* mice exhibit abnormal otolith function, while the SCCs remain unaffected. In contrast, *Zpld1^−/−^* mice exhibit impaired SCC function but normal otolith function. The *Zpld1* gene is primarily expressed in ampullary crista cells, and the *Zpld1* protein is a major component of the mouse cupula (Vijayakumar et al., 2019). Groups of five animals were used in each mutant group, with an additional wild-type group serving as a control.

### Data analysis

#### Pupil tracking

In this study, we employed a deep learning-based target recognition algorithm for pupil tracking. On this basis, threshold segmentation was applied, as depicted in Figure 1*C*. Each frame of the eye movement video was first processed through a pretrained YOLOv5 model to detect the pupil. The region containing the pupil was then cropped based on the predicted bounding box. Next, threshold segmentation was applied to convert the cropped image into a binary format. Finally, an ellipse-fitting algorithm was used to delineate the pupil contour, accurately determining the pupil's center. This method enabled precise tracking of the mouse pupil's displacement in both horizontal and vertical directions (Fig. 1*C4*,*C5*).

#### Fast-phase nystagmus extraction

In the pOVAR test, horizontal eye movements exhibit both FPN and SMC (Fig. 1*C4*), with their direction closely correlated to the motion of the eccentric axis. We implemented an algorithm from a previous study (Behrens and Weiss, 1992) to separate the fast phases and slow phases. First, the displacement data were processed using a low-pass filter to eliminate minor high-frequency vibrations. Subsequently, a second-order differentiation was applied to obtain the absolute acceleration values. FPN was identified in the time domain by applying appropriate thresholds, allowing for the separation of the fast and slow phases. Finally, the FPN frequency was calculated. Typically, we selected eye-tracking data collected 1 min after the device startup for analysis.

## Results

### The effects of peripheral (IDPN) and central disorder (baclofen) on FPN and sinusoidal modulation component in pOVAR test

The V-SMC during the pOVAR test on Day 9 after IDPN administration is shown in Figure 2*A*,*B*. The control group exhibited significantly larger displacement than both the mid-dose and high-dose groups across all tilt angles. Eye displacement in the mid-dose group (2 mg/g) was higher than that in the high-dose group (4 mg/g). The H-FPN frequency also displayed varying degrees of attenuation, but with considerable individual differences within groups, as shown in Figure 2*C*,*D* (two-way ANOVA). The horizontal mean slow-phase velocity/bias velocity was similar to the H-FPN frequency, as shown in Figure 2*E* (two-way ANOVA). These findings suggest that intraperitoneal IDPN injection damages the peripheral vestibular structures in mice. While SMC can be used for a quantitative assessment of vestibular dysfunction severity, H-FPN/bias velocity is also capable of quantifying vestibular damage, albeit with greater error than SMC, reflecting the complexity of FPN. Baclofen administration (5 and 10 mg/kg) had little effect on the modulation component during the pOVAR test (Fig. 2*G*,*H*), though the H-FPN frequency was reduced (Fig. 2*E*). Compared with the control group, the H-FPN frequency in the baclofen-treated groups was significantly reduced, with a more pronounced decrease in the high-dose (10 mg/kg) group (Fig. 2*F*, one-way ANOVA).

### UVL mice exhibit asymmetric FPN frequency in pOVAR test

**Figure 3.**
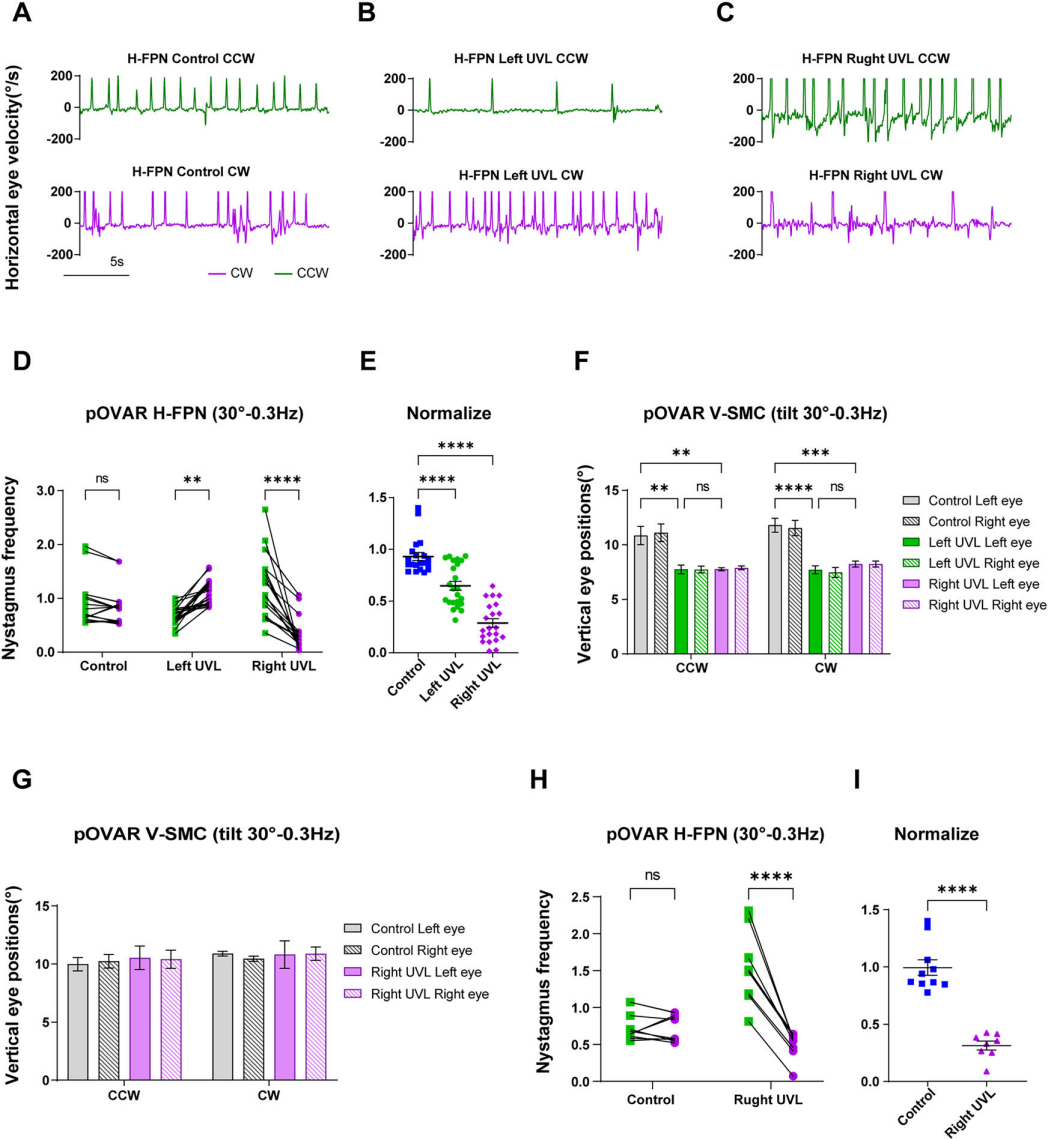
Dynamic characteristic of VOR in control, UVL, and unilateral SCD mice. Means ± standard error of mean (SEM) is shown in the results. ***A–C***, Traces show the horizontal eye velocity in healthy (control), left-UVL, and right-UVL mice during pOVAR test in both CCW and CW direction. ***D***, The H-FPN frequency in all groups during CCW and CW direction rotation (tilt angle: 30° at 0.3 Hz). ***E***, Normalize the data of graph (***D***). Control, CW/CCW; left-UVL, CCW/CW; right-UVL, CW/CCW. ***F***, Left and right eye V-SMC across all groups during CCW and CW stimulation (tilt angle: 30° at 0.3 Hz). ***G***, Similar to ***F***, V-SMC in control and L-SCD groups. ***H***, ***I***, Similar to ***D*** and ***E*** in right-UVL mice caused by L-SCD. After drilling a hole in the middle of LSC, the outflow endolymphatic fluid was absorbed with sterile cotton, after which the wound was sutured. The V-SMC and H-FPN frequency under other stimulus conditions are shown in Extended Data Figure 3-1 (*p < 0.05, **p < 0.01, ***p < 0.001, ****p < 0.0001).

During the pOVAR test on mice, the direction of the eccentric axis rotation was alternated (from CCW to CW) and kept opposite to the main axis rotation. Compared with the control group, V-SMC in the surgery group was significantly reduced in both CCW and CW directions. However, no significant difference was observed between the right-UVL and left-UVL groups. Additionally, no differences in V-SMC between the left and right eyes were detected under either CCW or CW stimulation conditions (Fig. 3*F*, two-way ANOVA). These results indicate that UVL surgery attenuates overall vestibular performance. However, using SMC data alone in the pOVAR test could not distinguish whether the injury occurred on the right or left side.

10.1523/ENEURO.0461-24.2025.f3-1 Figure 3-1 (**A, B, C**) Left and right eye V-SMC across all groups during CCW and CW stimulation (tilt angle: 20° at 0.3, 0.4  Hz, and 30° at 0.2  Hz). (**D**) The H-FPN frequency in all groups during CCW and CW directional rotation (tilt angle: 20° at 0.3  Hz). (**E**) Normalize the data of graph (D). Control: CW/CCW, left-UVL: CCW/CW, right-UVL: CW/CCW. (**F, G**) Similar to the graph (D, E), the tilt angle: 20° at 0.4  Hz. (**H, I**) Similar to (D, E), the tilt angle: 30° at 0.2  Hz. (**J**) Left and right eye V-SMC in control and L-SCD groups (tilt angle: 30° at 0.2  Hz). (**K, L**) Similar to the graph (D, E). [*p < 0.05, **p < 0.01, ***p < 0.001, ****p < 0.0001]. Download Figure 3-1, TIF file.

In contrast, the H-FPN frequency in left-UVL mice was significantly lower in the CCW direction than in the CW direction, while the opposite was true for right-UVL mice. No difference in H-FPN frequency between CCW and CW directions was detected in the control group (Fig. 3*A–E*; Extended Data Fig. 3-1*D–I*, two-way ANOVA). The normalized results are shown in Figure 3*E*. In the control group, the ratio of CW to CCW was ∼1, while in the surgery groups, the ratio was significantly <1, with the right-UVL group showing a more pronounced difference than the left-UVL group (one-way ANOVA). The V-SMC and H-FPN frequency under other stimulus conditions are shown in Extended Data Figure 3-1.

For mice with L-SCD and intact otolith function, V-SMC in the pOVAR test did not show significant differences compared with normal mice (Fig. 3*G*, Extended Data Fig. 3-1*J*). However, the H-FPN frequency also exhibited significant asymmetry (Fig. 3*H*,*I*; Extended Data Fig. 3-1*K,L*, two-way ANOVA). Therefore, the lesion side in UVL could be identified based on H-FPN, with the potential mechanism discussed in the discussion section.

**Figure 4.**
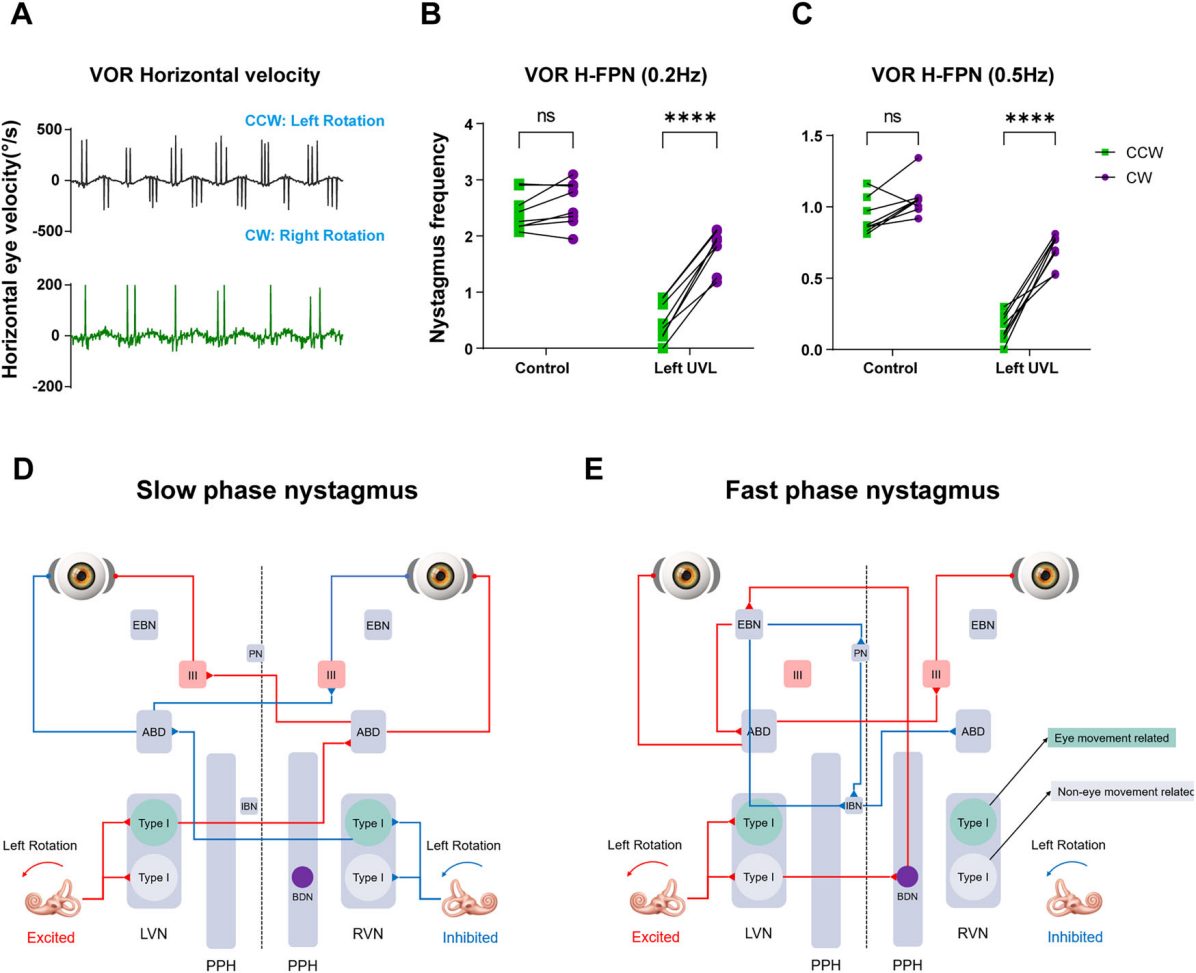
Illustration of the neural pathway for slow-phase and fast-phase eye movement in aVOR during leftward head rotation. ***A***, Traces show the horizontal eye velocity in control and left-UVL mice during aVOR test. ***B***, ***C***, the H-FPN frequency during aVOR test (0.2, 0.5 Hz) in control and left-UVL groups. ***D***, Neural pathway involved in SPN generation. ***E***, Neural pathway involved in FPN generation. LVN, left vestibular nucleus; RVN, right vestibular nucleus; PPH, prepositus hypoglossi; BDN, burster driver neuron; EBN, excitatory burst neuron; IBN, inhibitory burst neuron; PN, pause neuron; ABD, abducens neuron; III, ocular motor nucleus.

Furthermore, the FPN of UVL mice exhibited a distinct bias in aVOR test (Fig. 4*A*). During rotation toward the healthy side, normal FPN appeared; however, when rotating toward the injury side, the FPN significantly diminished or even ceased entirely (Fig. 4*B,C*). For slow-phase eye movements in the aVOR test, within certain limits, the bilateral H-SCCs generate symmetrical “push–pull” signals via the abducens nucleus and oculomotor nucleus, resulting in coordinated slow-phase movements (Fig. 4*D*). In contrast, the generation of FPN primarily relies on unilateral excitatory vestibular input, as shown in Figure 4*E* (Curthoys, 2002), thereby resulting in the bias of FPN.

10.1523/ENEURO.0461-24.2025.f4-1 Figure 4-1 (**A**) The H-FPN frequency during pOVAR test in control and *Zpld1^-/-^* mice (tilt angle: 20° at 0.2, 0.3, 0.4  Hz). (**B**) The V-SMC of control, *Zpld1^-/-^* and *Otop1^tlt/tlt^* mice during test (tilt angle: 20° at 0.2, 0.3, 0.4  Hz). (**C, D**) The V-SMC angle and gain value during OVAR test in CCW direction. (**E**) The H-FPN frequency during OVAR test in CCW direction. (**F**) The stimulus paradigm of the aVOR and TAV model. [*p < 0.05, **p < 0.01, ***p < 0.001, ****p < 0.0001]. Download Figure 4-1, TIF file.

### FPN reflects conditions on the SCC even in a traditional otolith testing mode

**Figure 5.**
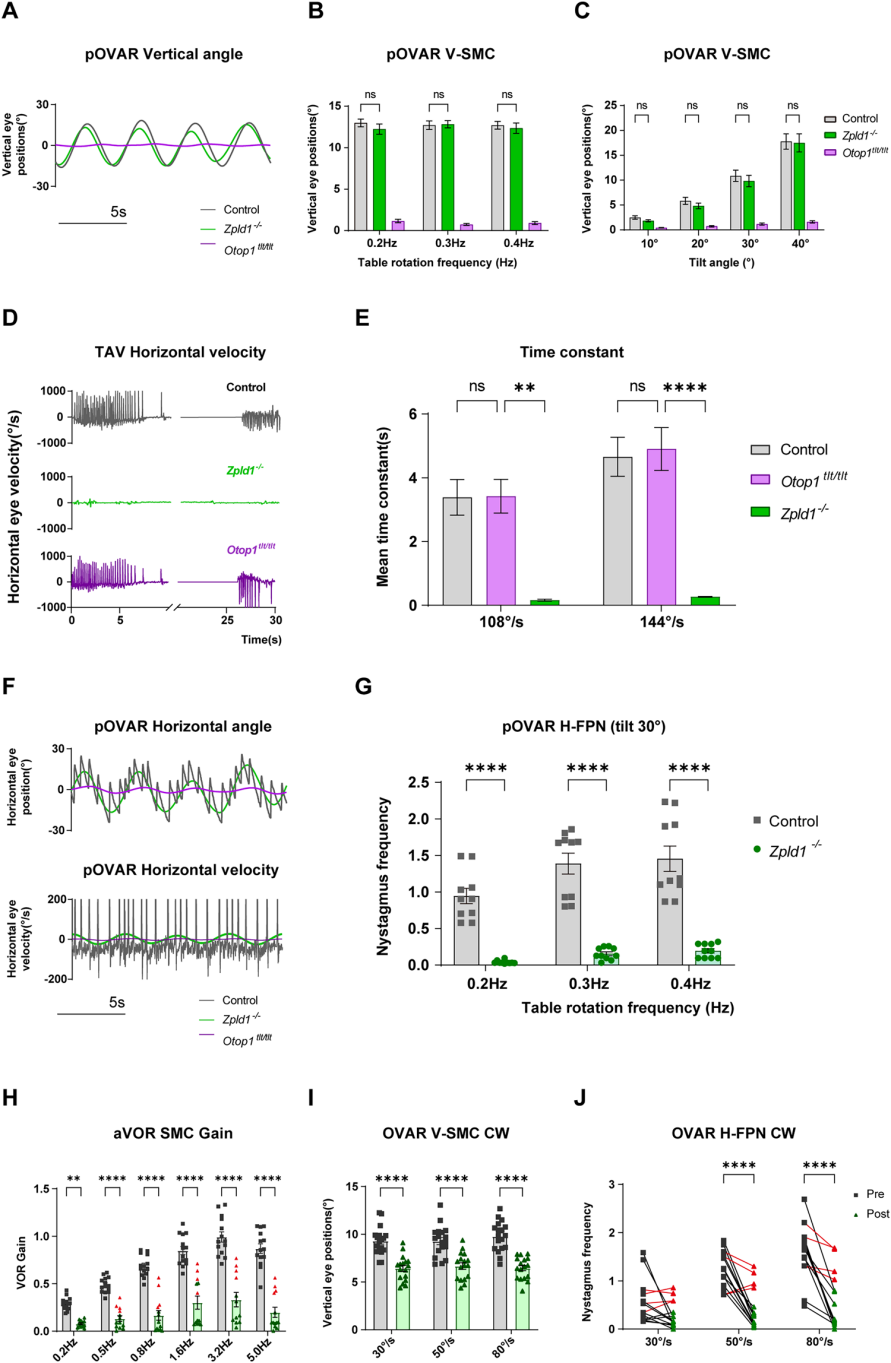
Vestibular function test in *Zpld1* and *Otop1* mutant mice. Means ± standard error of mean (SEM) is shown in the results. ***A***, Traces show vertical eye displacement during pOVAR test in control, *Zpld1^−/−^* and *Otop1^tlt/tlt^* mice. ***B***, V-SMC of control, *Zpld1^−/−^* and *Otop1^tlt/tlt^* mice during pOVAR test (tilt angle: 30° at 0.2, 0.3, 0.4 Hz). ***C***, V-SMC (tilt angle: 10°, 20°, 30°, 40° at 0.2 Hz). The V-SMC of control, *Zpld1^−/−^* and *Otop1^tlt/tlt^* mice during test (tilt angle: 20° at 0.2, 0.3, 0.4 Hz) are shown in Extended Data Figure 4-1*B*. ***D***, Traces show horizontal eye velocity during TAV test in control, *Zpld1^−/−^* and *Otop1^tlt/tlt^* mice. The stimulus paradigm of the TAV model is shown in Extended Data Figure 4-1*F*. ***E***, represent the vestibular time constant in TAV test. ***F***, Traces show horizontal eye displacement and horizontal eye velocity during pOVAR test in control, *Zpld1^−/−^* and *Otop1^tlt/tlt^* mice. ***G***, The H-FPN frequency during pOVAR test in control and *Zpld1^−/−^* mice (tilt angle: 30° at 0.2, 0.3, 0.4 Hz), and the results in other stimulus condition (tilt angle: 20° at 0.2, 0.3, 0.4 Hz) are shown in Extended Data Figure 4-1*A*. ***H***, ***I***, represent the SMC information of VOR and OVAR test in mice with bilateral L-SCD. ***J***, The H-FPN frequency during OVAR test in CW direction. The V-SMC gain and H-FPN frequency during OVAR test in CCW direction are shown in Extended Data Figure 4-1*C–E* (*p < 0.05, **p < 0.01, ***p < 0.001, ****p < 0.0001).

The *Otop1^tlt/tlt^* mice with otolithic dysfunction exhibited almost no VOR during the steady state of pOVAR test (Fig. 5*A–C*). However, at the onset and cessation of the test, nystagmus characterized by alternating fast and slow phases was observed due to the activity of the SCCs and VSM in the trapezoidal angular velocity (TAV) model (Fig. 5*D*). The stimulus paradigm of the TAV model was illustrated in Extended Data Figure 5-1*F*. *Otop1^tlt/tlt^* mice exhibited normal vestibular time constants, indicating the intact function of the SCCs and VSM. In contrast, *Zpld1^−/−^* mice demonstrated significant impairment in VSM function (Fig. 5*E*). The V-SMC results showed no difference between *Zpld1^−/−^* and WT mice under all stimulus conditions, indicating normal otolith function in *Zpld1^−/−^* mice (Fig. 5*B,C*; Extended Data Fig. 5-1*B*, two-way ANOVA). In contrast, there were significant differences in the horizontal fast-phase component, with *Zpld1^−/−^* mice lacking H-FPN (Fig. 5*F,G*; Extended Data Fig. 5-1*A*, two-way ANOVA). Bilateral L-SCD surgery in mice resulted in inactivation of the H-SCCs, a significant decrease in VOR gain value, and a slight impairment of otolith function (Fig. 5*H,I*; Extended Data Fig. 5-1*C,D*, two-way ANOVA). Due to surgical variability, some mice retained higher levels of SCC function, as indicated by the red triangles in Figure 5*H,J*. Notably, bilateral H-SCCs dysfunction led to a significant reduction in H-FPN during the OVAR test (Fig. 5*J*; Extended Data Fig. 5-1*E*, two-way ANOVA). These results indicated that the input from the SCCs may contribute to the generation of the H-FPN during pOVAR and OVAR tests.

10.1523/ENEURO.0461-24.2025.f5-1 Figure 5-1 Download Figure 5-1, TIF file.

## Discussion

### The signals from SCCs can influence the generation of FPN in mouse OVAR test

During the pOVAR test, nystagmus is induced as long as rotation persists, consisting of SMC modulated at the same frequency as the stimulus and bias component related to horizontal eye velocity (Kushiro et al., 2002). The SMC exhibits a high degree of consistency with vestibular stimulation. V-SMC reflects changes in head orientation relative to gravity, whereas H-SMC reflects the effect of translational acceleration acting on the head. It is widely accepted that the bias component established by VSM to compensate for head angular velocity in the OVAR test originates from the activation of the otolith organs (Darlot et al., 1988). The central VSM, acting as a multisensory rotational estimator, integrates rotational information through its internal rotational sensors to decompose ambiguous otolith signals into tilt and translation components. As the SCCs signals decay, the emergence of bias allows the VSM to maintain a constant velocity output, thereby minimizing otolith prediction errors to the greatest extent (Laurens and Angelaki, 2011). In practice, the bias component opposite to head movement is equivalent to the H-FPN in the same direction as the head, due to the eye movements within the orbit. Consequently, the H-FPN frequency accurately reflects the magnitude of the bias component. From a data processing perspective, calculating the bias component typically introduces larger errors compared with extracting FPN components.

**Figure 6.**
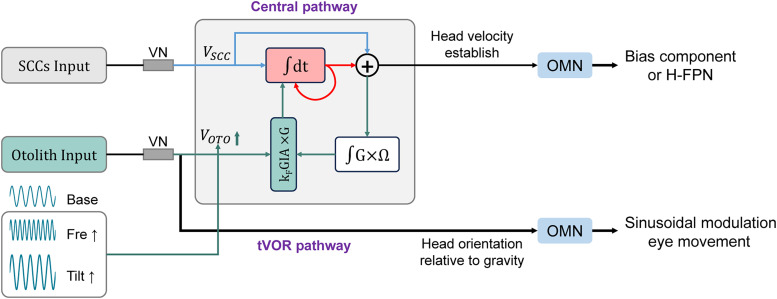
Schematic diagram of direct and indirect pathways for SCCs and otolith information in the OVAR test. VN, vestibular nucleus; OMN, ocular motor neurons; *V*_SCC_, SCCs velocity signal; *V*_OTO_, otolith signal; GIA, gravito-inertial acceleration; *k_F_*, coefficient; Ω, angular velocity vector; G, gravity vector; ×, vectorial cross-product; ∫G × Ω, tilt estimator.

However, *zpld1^−/−^* mice with intact otolith function exhibit no FPN component during the OVAR test, indicating that the SCCs may also contribute to the generation of FPN. This hypothesis is further supported by results from mice with bilateral L-SCD, which corroborate this perspective. Mice with only unilateral semicircular canal injury also exhibited abnormalities in the FPN. Additionally, *Otop1^tlt/tlt^* mice exhibit a normal vestibular time constant, indicating that both SCCs and VSM functions are intact (Khan et al., 2019). At the onset of OVAR, the SCCs are activated, leading to the appearance of “jerk” nystagmus. As the rotation continues, the SCCs signals gradually decay to resting levels (Raphan et al., 1983), and in the absence of otolith input, both SMC and FPN disappear. The SCCs signals serve as the primary input to the central VSM rotational velocity estimator. Any form of SCC dysfunction can impair the central establishment of head angular velocity (Denise et al., 1996). Consequently, bilateral semicircular canal damage may result in the loss of the VSM's ability to estimate angular head velocity. The emergence of the bias component or FPN serves as a compensatory mechanism for the decline in SCC input, resulting from internal tilt estimator feedback to the VSM. Crucially, through this feedback loop, the central nervous system can convert the continuous otolithic signals into a constant angular velocity (Fig. 6, k_F_GIA × G; Laurens and Angelaki, 2011). In summary, the generation of FPN in the OVAR model requires continuous otolithic signals, while the VSM converts periodic oscillatory otolithic inputs into a sustained FPN (bias component). Otolith signals are transduced into V-SMC via the orientation mechanisms. Additionally, increasing the rotational frequency or the tilt angle both lead to an increase in *V*_OTO_, ultimately resulting in an increase in H-FPN frequency.

### The FPN effectively identifies a variety of subtle vestibular dysfunctions

The generation of FPN in OVAR relies on peripheral otolith input and central VSM, implying that abnormalities in FPN may indicate dysfunctions in either the peripheral or central pathways. If the injury affects only the central pathway, the FPN is affected while the SMC remains relatively normal, as observed following baclofen injections (Shimizu et al., 2016). However, both the FPN and SMC can be affected if the injury involves peripheral receptors, such as in the case of IDPN administration or UVL induced by gentamicin injections.

In this study, the bias in FPN exhibits high sensitivity for detecting unilateral vestibular impairment, even after vestibular compensation has occurred. This bias is attributed to asymmetric oscillatory signals from the peripheral labyrinth being transmitted to the central velocity storage network. In clinical practice, the diagnosis of central vestibular lesions using head-shaking-induced nystagmus relies on a similar mechanism (Choi and Kim, 2009). Some studies have shown that the brain optimizes the velocity storage integration network based on the signal-to-noise ratio (SNR) of peripheral vestibular input (Madhani et al., 2022). Even following central compensation after peripheral damage, the noise inherent in lesioned afferents and vestibular nuclei persists, leading to a reduction in the SNR of ipsilateral input signals. This reduction results in a decreased vestibular time constant and diminished capacity of central VSM to reconstruct head angular velocity. Consequently, during the OVAR test, there is a reduction in either the bias component or the FPN.

### Summary

In this study, we investigated the contribution of different vestibular structures to the generation of FPN during the OVAR test in mice. Our findings align with previous research on primates, demonstrating that the generation of FPN in the OVAR test requires not only otolithic signals but also substantial input from the SCCs. The SCCs provide the primary input to the neural estimator of the rotational velocity in the central VSM, and the reconstruction of angular velocity using otolith signals by the central nervous system also depends on the participation of this estimator. Consequently, SCC input may modify this rotation estimator to regulate the generation of FPN during OVAR and explain the FPN bias following UVL. Additionally, the FPN frequency during OVAR was extracted, facilitating the effective identification of subtle vestibular dysfunctions, particularly those undetectable using traditional SMC assessments. For instance, the central pathways involving the VOR, such as the vestibulocerebellum, as well as UVL, align with previous findings (Gilchrist et al., 2003; Fig. 4*C*) and exhibit a more pronounced contrast.

## Synthesis

Reviewing Editor: David Schoppik, New York University Grossman School of Medicine Decisions are customarily a result of the Reviewing Editor and the peer reviewers coming together and discussing their recommendations until a consensus is reached. When revisions are invited, a fact-based synthesis statement explaining their decision and outlining what is needed to prepare a revision will be listed below. The following reviewer(s) agreed to reveal their identity: Jean Laurens, Jerome Carriot. Note: If this manuscript was transferred from JNeurosci and a decision was made to accept the manuscript without peer review, a brief statement to this effect will instead be what is listed below.

Major remarks:

1. The authors contrast slow-phase nystagmus (SPN) and fast-phase nystagmus (FPN), drawing conclusions such as FPN's dependence on the velocity storage mechanism. However, this distinction is misleading. In reality, they are comparing vertical SPN to horizontal FPN, and the pertinent comparison should be vertical versus horizontal, not SPN versus FPN.

The authors' statement in line 315 supports this point: "In practice, the bias component in the direction opposite to head movement is equivalent to the H-FPN in the same direction as the head, due to the movement of the eyes within the orbit. Consequently, the H-FPN firing rate accurately reflects the magnitude of the bias component. From a data processing perspective, calculating the bias component tends to introduce larger errors compared to extracting FPN components."

This clearly indicates that horizontal SPN and FPN are equivalent, and that the difference is primarily a data processing issue. This issue is likely related to the signal-to-noise ratio and may be due to the technical difficulty of measuring eye movements in mice. Horizontal SPN can be measured accurately in primates, particularly using search coil techniques. In fact, the results of this study regarding horizontal FPN, and how it is affected by canal lesions, are consistent with previous findings in non-human primates (NHPs), where horizontal SPN was measured.

Moreover, referring to vertical SPN in the context of this manuscript may be incorrect. The vertical eye movements described here appear to be an orienting vestibulo-ocular reflex-a modulation of eye position in response to head tilt, where the brain tends to align the gaze axis with the horizontal plane. This is not the same as nystagmus, which is driven by an eye velocity command-the eyes rotate at a certain velocity, reach the limit of the orbit, and then reset their position through a saccade. If the vertical eye movements are not nystagmus, then the term SPN should not be used.

In summary, while the results of the manuscript are essentially correct, the authors should focus on comparing horizontal versus vertical eye movements, rather than contrasting SPN with FPN. Additionally, they should be more specific regarding the type of eye movements recorded here-i.e., horizontal angular VOR (driven by the velocity storage mechanism, as correctly stated by the authors) versus vertical otolith-driven orienting reflex.

Finally, it would be helpful if the authors could present the velocity of the horizontal bias. While we understand that it may be harder to measure in their experiments, this would be very valuable data for other labs, especially for modelling studies where model predictions can be directly compared to SPN.

2. One of the main conclusions of the study is that semicircular canal lesions abolish horizontal nystagmus during OVAR, presumably through the loss of velocity storage. This finding is well established: many studies in NHPs have demonstrated that velocity storage is abolished following canal lesions. This effect is apparent during OVAR, as shown in the current study, and has also been observed through the loss of optokinetic after-nystagmus, another velocity-storage-dependent mechanism. See:

https://doi.org/10.1016/0006-8993(83)90558-9

https://doi.org/10.1111/j.1749-6632.1996.tb15769.x

https://link.springer.com/article/10.1007/s002210000364

https://doi.org/10.1111/j.1749-6632.1992.tb25216.x

There are also theoretical arguments explaining why canal lesions affect velocity storage, as outlined by Madhani et al. (2022) and in previous publications

(e.g. https://doi.org/10.1152/jn.90677.2008).

3. OVAR is a type of stimulation in which the subject is spun at a constant velocity around an axis that is not aligned with the vertical (gravity). Two key parameters are crucial in this context:

1) Alignment of the vestibular system with the axis of rotation: Accurate alignment is essential to ensure that the stimulation and resulting measurements are valid.

2) Timing: With a time constant of approximately 20 seconds, the semicircular canals stop responding after about 1 minute of constant velocity (three times the time constant). Consequently, the angular VOR (vestibulo-ocular reflex) follows a similar trend. However, because the axis of rotation is not aligned with gravity, the head undergoes continuous reorientation relative to gravity, stimulating the otolith organs and resulting in a more complex VOR, including counter-rolling, vertical, and horizontal movements. Then, depending on the timing, the VOR will not have the same origin.

Instead of tilting their apparatus relative to the gravity, the authors use a centrifuge in order to produce a radial acceleration that coupled with the gravity will produce a new tilted reference: the gravito-inertial acceleration. While this approach is innovative, it relies heavily on precise alignment of the vestibular system with the axis of rotation. Without this alignment, the angle of the gravito-inertial acceleration would change continuously, introducing variability into the results. These two critical points-the precise alignment of the vestibular system and the timing of measurements-are not clearly addressed in the manuscript. Without this clarity, it is challenging to evaluate the validity of the results and conclusions.

4. Lines 311-315: "Experimental evidence suggests that neither otolith nor canal afferents exhibit a bias state, implying that the bias component originates from the vestibular center (Goldberg &Fernandez, 1982; Raphan et al., 1983). Consequently, it is widely accepted the bias component, which compensates for the head angular velocity during OVAR test and is established by VSM, arises from the activation of the otolith organs (Darlot et al., 1988)." The literature and concepts here are somewhat outdated. The current view on bias velocity during OVAR is that central vestibular pathways attempt to predict the periodic activation of the otoliths based on an internal sensation of rotation, and that bias velocity (reflecting the true rotation of the head) emerges as a solution to minimize otolith prediction errors. See for instance Merfeld's model (https://doi.org/10.3109/00016489509125269, or Laurens and Angelaki 2011).

5. Figure 5: Direct versus indirect pathways, and the associated text. There seems to be a misinterpretation of the classical concepts of direct and indirect pathways. In the classical Raphan-Cohen model (e.g. https://link.springer.com/article/10.1007/BF00236613 ), the direct and indirect pathways are defined with relation to angular VOR only. Specifically, the indirect pathway refers to the velocity storage, and the direct pathway as an additional, additive contribution of SCC and retinal flow. Importantly, there is no direct otolith contribution to the angular VOR.

The vertical otolith-driven eye movements described here belong to a different process: the orienting otolith-ocular reflex. They are not part of the angular VOR circuit. Therefore, referring to a 'direct' otolith pathway and presenting it as shown in Figure 5 is misleading, and we recommend revising this representation.

6. Line 330-332: "Crucially, at the initiation of OVAR, the SCCs signal (VSCC) must first be transmitted to the VSM, acting as a primer to ensure the continuous and accurate conversion of otolith signals.". This statement is incorrect. If you 'initiate' OVAR without activating the SCC, bias velocity will still develop and reach the same level as if the SCC were activated. This can be shown:

- by the experiments described in the current manuscript, if the 'inner axis' and 'external axis' velocities are exactly opposite. In this situation, the bias velocity will develop, even though the canals are never activated.

- by performing an OVAR as in https://doi.org/10.1016/0006-8993(83)90558-9 , Fig. 1D, or https://doi.org/10.1152/jn.00143.2010 , Fig. 1D: rotation starts with the head (and the rotation axis) upright, allowing the canal response to decrease, followed by tilting the rotation axis.

7. Line 337-354: We disagree with this interpretation. First, as mentioned above, there is no difference between horizontal SPN and FPN. Second, how can the FPN depend from 'impulses from the H-SCC transmitted to type I neurons, and then BDN and PPN', since the SCC are not activated during OVAR. The simplest explanation for the asymmetry following a unilateral lesion is that velocity storage has been modified asymmetrically.

8. Lines 357-358: "If the injury involves only the indirect pathway of the VOR, FPN is affected while SPN remains normal": once again, this conflates horizontal and vertical eye movements. An injury to the indirect pathway would affect both horizontal SPN and horizontal FPN equally.

9. Data presentation: The strength of the manuscript lies in the large amount of data collected by the authors. Unfortunately, the presentation of the data is at times somewhat disorganized. For instance, in Fig. 2, the authors present raw traces of vertical eye position velocity (panel A), histograms of mean eye movements (panels B, C), followed by raw horizontal velocity but not position, now in three separate panels, and then raw eye velocity focusing on the baclofen group. This inconsistent presentation makes the manuscript difficult to follow and results in missing important data for the reader. Could the authors present the data in a more complete and structured manner?

Minor remarks:

Firing rate: The term "firing rate" specifically refers to the rate at which neurons fire action potentials. It is not related to the fast phase of nystagmus, as suggested in the manuscript.

Line 74: Be careful, OVAR is not the same as pitch and roll oscillations, which would activate the vertical canals. This distinction has been the topic of previous studies, e.g. https://doi.org/10.1016/0006-8993(87)91659-3 (Fig. 2b versus Fig. 2a) and https://doi.org/10.1523/JNEUROSCI.5900-10.2011 (also Fig. 2b versus Fig. 2a).

Line 76: "The OVAR test is used to measure otolith function and is not sensitive to canal function deficits": As explained above, this is incorrect since canal function deficits decrease or abolish velocity storage.

Line 160: Could you express velocities in degrees per second, which are generally used in vestibular literature, rather than rpm?

Lines 232 to 249: How did you calibrate the eye trackers? How do you extract and use the corneal reflection?

Fig. 2A: The vertical eye movements in the 2 mg/g group are clearly out of phase compared to controls. How do you explain this?

Around line 259: Please explain Fig. 2D and 2E in the text.

Line 274: Be more explicit: your conclusion is that UVL destroys both the canals and otoliths, correct?

Line 292: You never show the nystagmus at the onset of rotation, nor discuss its time constant. Do you observe any clear velocity storage activity?

Line 334: Yes, this has been shown in NHPs by Kushiro et al. (2002), Fig. 2, and https://link.springer.com/article/10.1007/s002210000364, Fig. 4.

When you compute the FPN, do you take the direction of the saccades into account? For instance, if the mouse performs 10 saccades to the left in a given period, this is not the same as performing 5 saccades to the left and 5 to the right.

In addition to the eye movements discussed so far, there is a periodic modulation of horizontal eye position, evident in Fig. 1.C.4 and Fig. 4A. Could this be attributed to an otolith-driven orienting reflex affecting horizontal eye position? Additionally, if the authors record both eyes, could they provide some representative binocular traces? It would be valuable to see whether the two eyes are in phase or out of phase.
