# Supplementary figures and images for: Semicircular Canals Input Can Modify the Fast-Phase Nystagmus in Off-Vertical Axis Rotation of Mice
Source: eNeuro. 2025 Mar 4;12(3):ENEURO.0461-24.2025. doi: 10.1523/ENEURO.0461-24.2025 (PMC11963835; doi:10.1523/ENEURO.0461-24.2025)

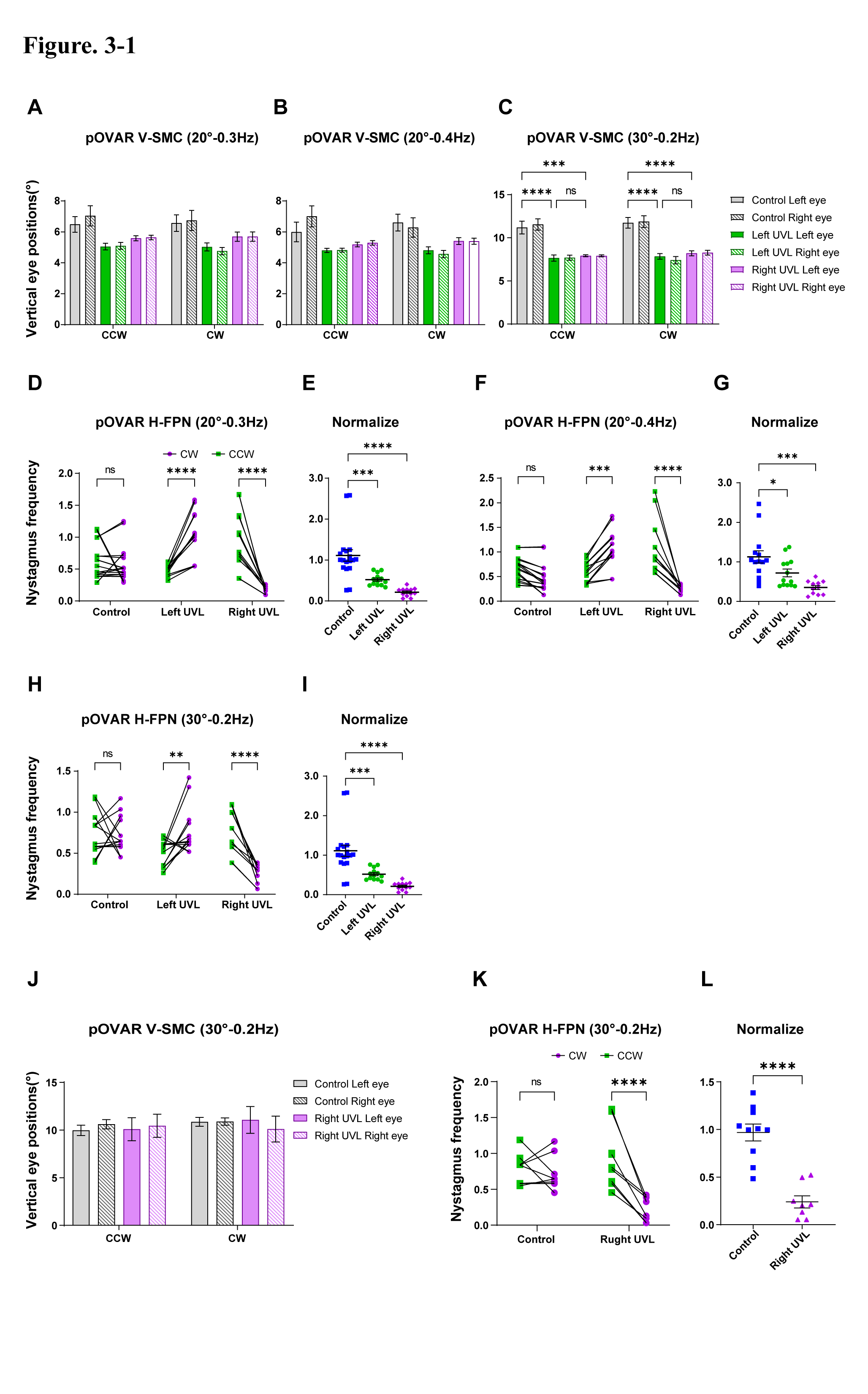

Supplement: Figure 3-1 — (A, B, C) Left and right eye V-SMC across all groups during CCW and CW stimulation (tilt angle: 20° at 0.3, 0.4 Hz, and 30° at 0.2 Hz). (D) The H-FPN frequency in all groups during CCW and CW directional rotation (tilt angle: 20° at 0.3 Hz). (E) Normalize the data of graph (D). Control: CW/CCW, left-UVL: CCW/CW, right-UVL: CW/CCW. (F, G) Similar to the graph (D, E), the tilt angle: 20° at 0.4 Hz. (H, I) Similar to (D, E), the tilt angle: 30° at 0.2 Hz. (J) Left and right eye V-SMC in control and L-SCD groups (tilt angle: 30° at 0.2 Hz). (K, L) Similar to the graph (D, E). [*p < 0.05, **p < 0.01, ***p < 0.001, ****p < 0.0001]. Download Figure 3-1, TIF file. [file eneuro-12-ENEURO.0461-24.2025-s001.tif]

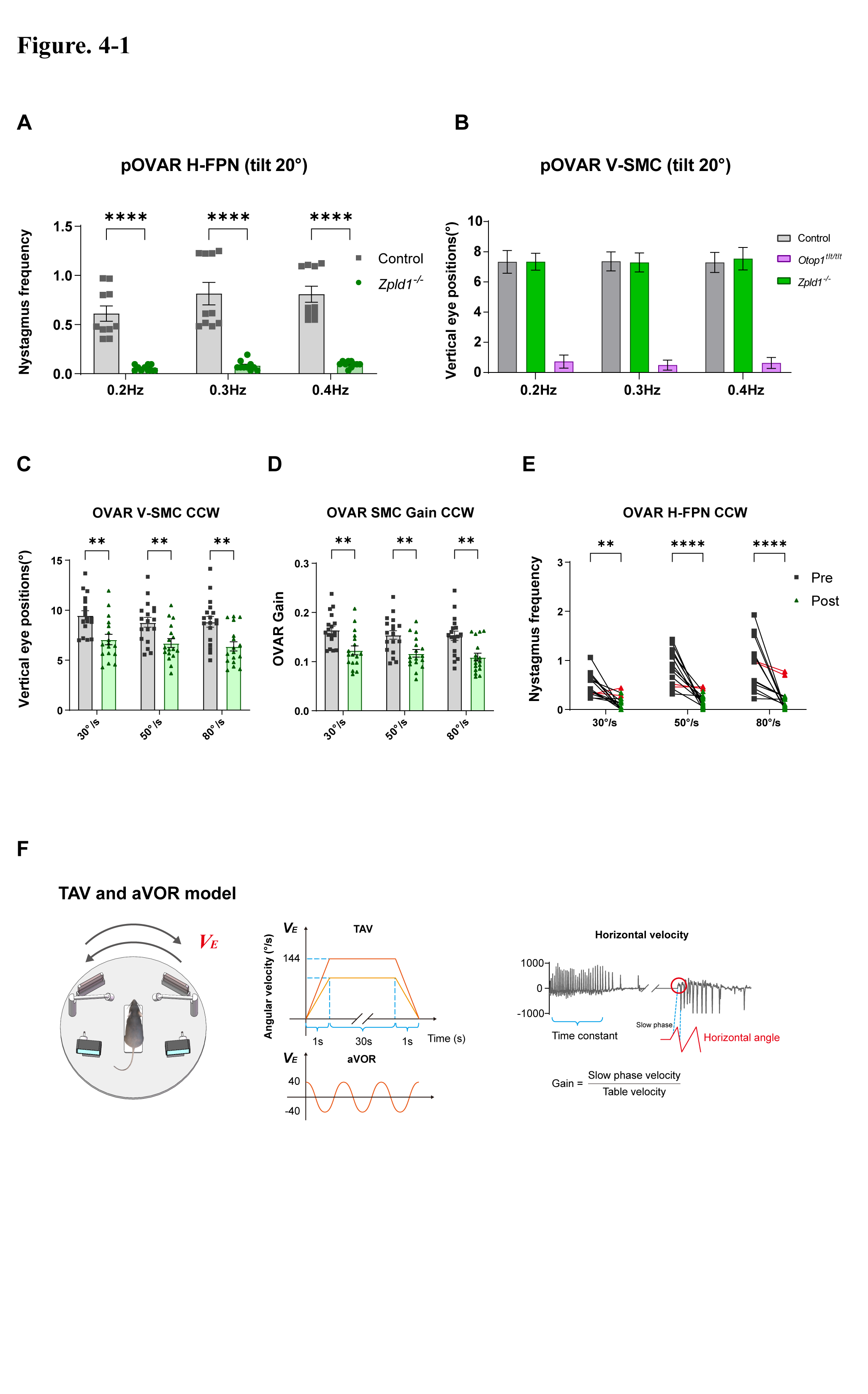

Supplement: Figure 4-1 — (A) The H-FPN frequency during pOVAR test in control and Zpld1-/- mice (tilt angle: 20° at 0.2, 0.3, 0.4 Hz). (B) The V-SMC of control, Zpld1-/- and Otop1tlt/tlt mice during test (tilt angle: 20° at 0.2, 0.3, 0.4 Hz). (C, D) The V-SMC angle and gain value during OVAR test in CCW direction. (E) The H-FPN frequency during OVAR test in CCW direction. (F) The stimulus paradigm of the aVOR and TAV model. [*p < 0.05, **p < 0.01, ***p < 0.001, ****p < 0.0001]. Download Figure 4-1, TIF file. [file eneuro-12-ENEURO.0461-24.2025-s002.tif]

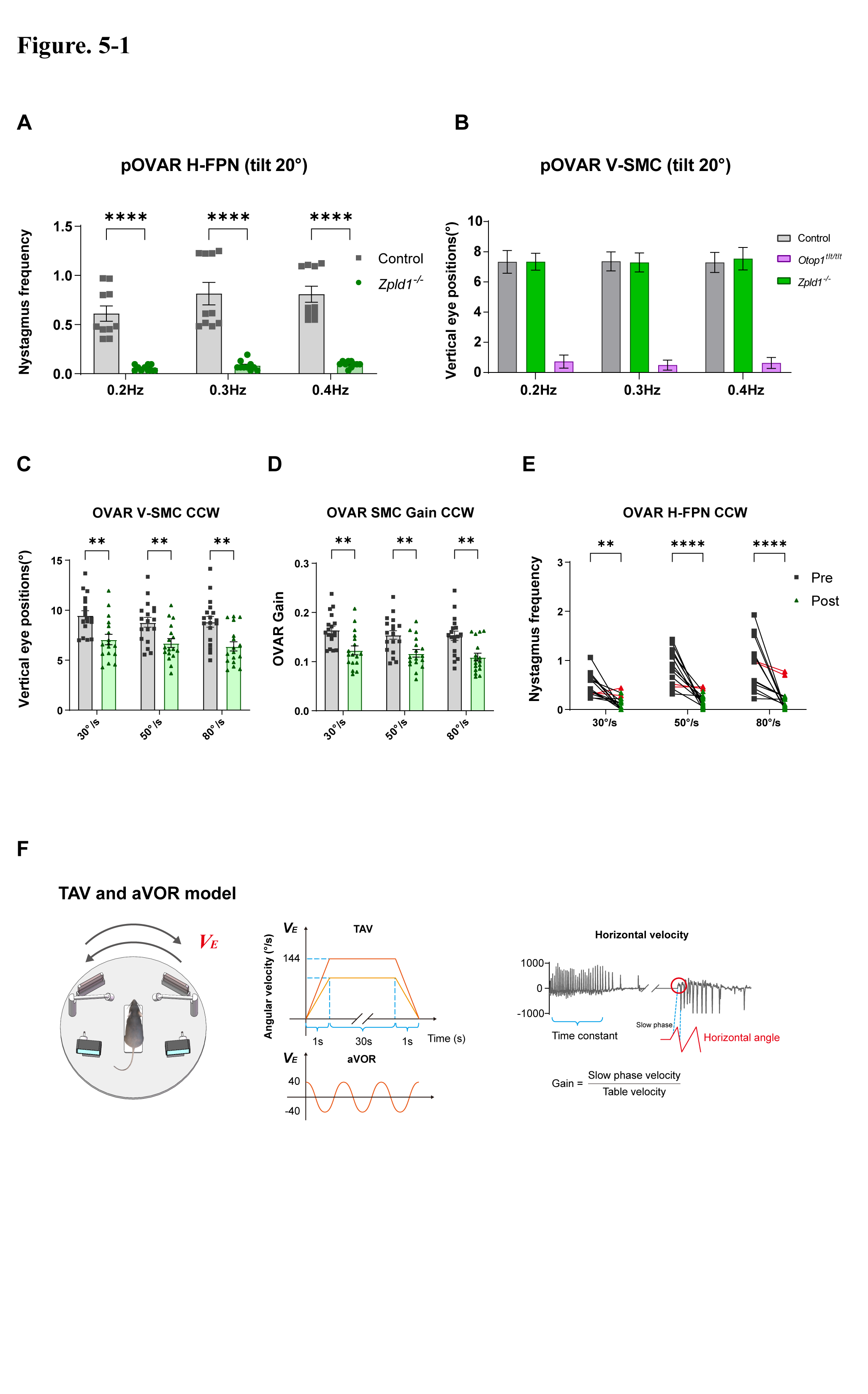

Supplement: Figure 5-1 — Download Figure 5-1, TIF file. [file eneuro-12-ENEURO.0461-24.2025-s003.tif]
